# Hydraulic segmentation explains differences in loss of branch conductance caused by fire

**DOI:** 10.1093/treephys/tpad108

**Published:** 2023-09-06

**Authors:** Adam G West, Shonese T Bloy, Robert P Skelton, Jeremy J Midgley

**Affiliations:** Department of Biological Sciences, University Avenue, University of Cape Town, 7701, Cape Town, South Africa; Department of Biological Sciences, University Avenue, University of Cape Town, 7701, Cape Town, South Africa; SAEON Fynbos Node, Centre for Biodiversity Conservation, Kirstenbosch Gardens, 7708, Cape Town, South Africa; Animal, Plant and Environmental Sciences, University of the Witwatersrand, 1 Jan Smuts Ave, Braamfontein, 2001, Johannesburg, South Africa; Department of Biological Sciences, University Avenue, University of Cape Town, 7701, Cape Town, South Africa

**Keywords:** cavitation, embolism, *Eucalyptus cladocalyx*, hydraulic conductance, hydraulic failure

## Abstract

The hydraulic death hypothesis suggests that fires kill trees by damaging the plant’s hydraulic continuum in addition to stem cambium. A corollary to this hypothesis is that plants that survive fires possess ‘pyrohydraulic’ traits that prevent heat-induced embolism formation in the xylem and aid post-fire survival. We examine whether hydraulic segmentation within stem xylem may act as such a trait. To do so, we measured the percentage loss of conductance (PLC) and vulnerability to embolism axially along segments of branches exposed to heat plumes in two differing species, fire-tolerant *Eucalyptus cladocalyx* F. Muell and fire-sensitive *Kiggelaria africana* L., testing model predictions that fire-tolerant species would exhibit higher degrees of hydraulic segmentation (greater PLC in the distal parts of the branch than the basal) than fire-intolerant species (similar PLC between segments). Following exposure to a heat plume, *K. africana* suffered between 73 and 84% loss of conductance in all branch segments, whereas *E. cladocalyx* had 73% loss of conductance in whole branches, including the distal tips, falling to 29% in the most basal part of the branch. There was no evidence for differences in resistance segmentation between the species, and there was limited evidence for differences in distal vulnerability to embolism across the branches. Hydraulic segmentation in *E. cladocalyx* may enable it to resprout effectively post-fire with a functional hydraulic system. The lack of hydraulic segmentation in *K. africana* reveals the need to understand possible trade-offs associated with hydraulic segmentation in long-lived woody species with respect to drought and fire.

## Introduction

Fire is an important ecological driver in many ecosystems, influencing vegetation structure and function, biome distribution, biodiversity and productivity (Fensham et al. 2003, Bond and Keeley 2005). Fire exposes trees to severe biophysical stressors that include direct combustion of living material, convective, conductive and radiative transfer of heat damaging living tissues, and the creation of a dry heat plume that may desiccate the canopy (Michaletz and Johnson 2007, Kavanagh et al. 2010, Midgley et al. 2011, Michaletz et al. 2012, West et al. 2016, Hood et al. 2018, Bär et al. 2019). Trees that are well protected from heat damage through thick bark, or well-protected buds, may emerge post-fire with living tissues intact (Lawes et al. 2011*a*, 2011*b*). However, unless the trees also emerge post-fire with intact hydraulic systems that can support transpiration and gas exchange, these tissues may rapidly desiccate and die. This hydraulic mechanism of post-fire mortality, the hydraulic death hypothesis (Midgley et al. 2011), was formulated based on field observations of rapid post-fire death of unburnt tissues which was more rapid than expected due to cambial necrosis or damage to upstream phloem (Michaletz and Johnson 2007, Midgley et al. 2011, Michaletz et al. 2012). Additional evidence from fire heat plume modeling (Kavanagh et al. 2010), experimental studies (Michaletz et al. 2012, West et al. 2016) and field observations (Bär et al. 2019) have demonstrated the potential for fire-induced hydraulic failure to occur in the xylem.

Hydraulic failure in the xylem post-fire may occur through direct heating-induced impacts on the xylem, resulting in thermal softening of lignin and subsequent conduit collapse or deformation (Michaletz et al. 2012, West et al. 2016). Hydraulic failure may also occur through indirect effects of heating on the plant that result in embolism formation by air-seeding modeling (Kavanagh et al. 2010). Under normal conditions of water stress, plants minimize embolism formation in the xylem by closing stomata to halt water loss through transpiration. However, heat plumes above fires rapidly expose trees to hot and dry conditions (i.e., high vapor pressure deficit) that may induce rapid water loss from the tree, causing a very high tension in the xylem sap, which may induce embolism formation through air-seeding (Kavanagh et al. 2010). Recent evidence suggests that an accelerated rate of water loss during a heat plume may be a biophysical inevitability for trees (Hoffmann et al. 2021), thus demanding a biological solution to mitigate this problem of potential hydraulic dysfunction.

Using an experimental approach, West et al. (2016) demonstrated considerable loss of hydraulic conductance in distal shoots in two species of trees, differing in fire-tolerance, exposed briefly to hot, dry air at two different temperatures. At the higher temperature, both species experienced similarly high percentage loss of conductance (PLC; 80%). However, surprisingly, the fire-tolerant species experienced a greater PLC than the fire-intolerant species at the lower temperature despite having more embolism-resistant xylem (West et al. 2016). Model simulations could reproduce the experimental results by assuming a greater vulnerability to embolism in the distal parts of the shoot of the fire-tolerant species, but not in the fire-intolerant species. It was thus hypothesized that some form of hydraulic segmentation may be an important ‘pyrohydraulic’ trait enabling post-fire survival (West et al. 2016).

In this paper, we define ‘hydraulic segmentation’ as the loss of conductance being localized to the distal portions of the hydraulic continuum, thereby protecting the remainder of the hydraulic continuum from hydraulic failure. This usage is consistent with West et al. (2016) and is analogous to the concept of the ‘hydraulic fuse’ (Tyree and Zimmermann 2002). Such segmentation may enable a tree to localize the emboli to distal expendable organs, protecting larger organs that are a major carbon investment that take years to grow (Tyree and Ewers 1991, Tyree and Zimmermann 2002) and from which they could resprout.

Hydraulic segmentation could come about through two distinct, but non-exclusive, mechanisms. The first is resistance segmentation. Resistance segmentation occurs when there is an increase in hydraulic resistance in the distal portions of the hydraulic pathway that can, for a given flow rate, result in an increased water potential gradient across the tissue in question (Zimmermann 1978, Tyree and Zimmermann 2002). With resistance segmentation, water potential gradients could result in varying spatial patterns of embolism even with similar xylem vulnerability to embolism. The second mechanism is vulnerability segmentation, where there is a difference in the vulnerability to embolism of the distal tissue compared with more basal tissues, presumably due to anatomical- or structural-related changes to the xylem (Tyree and Ewers 1991, Tyree and Zimmermann 2002). In a recent work, this has been referred to as safety segmentation (Mayr 2021). Resistance and vulnerability segmentation can combine, or operate independently (Levionnois et al. 2020), but both result in the phenomenon of hydraulic segmentation. Evidence for hydraulic segmentation under drought conditions has been noted, showing that, where it occurs, embolism is more likely to occur in distal shoots, petioles or leaves (Tyree and Sperry 1989, Johnson et al. 2009, Bucci et al. 2012, 2013, Pivovaroff et al. 2014, Levionnois et al. 2020). Hoffmann et al. (2021) demonstrated acute water loss from cut shoots of *Magnolia grandiflora* exposed to leaf combustion in a lab experiment. They inferred that hydraulic segmentation did not prevent acute water loss in this species. However, hydraulic segmentation was neither measured directly in this study nor was it determined how it might vary along the length of the stem, or between species with different fire survival abilities. As such, the role of hydraulic segmentation as a ‘pyrohydraulic trait’, influencing post-fire survival, needs further exploration.

In this paper, we test the hypothesis of West et al. (2016) that the fire-tolerant species *Eucalyptus cladocalyx* F. Muell, experiences a greater degree of hydraulic segmentation than the fire-intolerant species *Kiggelaria africana* L. when exposed to a heat plume. Using the same two species and the same heat-plume simulation methods as West et al. (2016), we measured PLC in progressively trimmed shoots to test if PLC was concentrated distally. Additionally, we measured the vulnerability to embolism at distal and more basal parts of the shoots using the optical vulnerability method. We hypothesized that *E. cladocalyx* would have a greater degree of PLC distally than basally, whereas this would not be the case for *K. africana*. Further more, we predicted that this hydraulic segmentation in *E. cladocalyx* would be due to an increase in vulnerability to embolism in the distal shoot, whereas *K. africana* would have no difference in vulnerability between sections.

## Materials and methods

### Plant material

As per West et al. (2016), we selected tree species with differing fire strategies, a fire-tolerant species—*E. cladocalyx* F. Muell, and a fire sensitive species—*K. africana* L. Branch samples, ~2 m in length, were collected from trees on the University of Cape Town grounds (33.96S, 18.46E). Maximum xylem vessel length for *E. cladocalyx* and *K. africana* were previously determined as 61 and 56 cm, respectively (West et al. 2016) and therefore our branches far exceeded the maximum vessel length. Stem xylem water potentials of the collected branches were measured (Scholander-type Pressure Chamber; PMS Instrument Company Model 1505D) to ensure branches were not water-stressed upon collection and were typically >−0.3 MPa. Once cut, the ends of the cut branches were immediately placed in water and were transported to the lab. In the lab, branches were re-cut under water, ensuring they exceeded 65 cm from the cut end. These branches were then subject to two different experimental measurements: xylem conductance measurements, following the methodology of West et al. (2016); and assessment of vulnerability to embolism using the optical technique (Brodribb et al. 2017).

### Experimental design

Branches, with the cut end placed in water, were left to acclimatize for 4 h under a hood light. This ensured stomata were open, and transpiration was taking place. Control and treatment branches were then trimmed under water to c. 65 cm from the distal tip. The dripping wet cut end was then wrapped in Parafilm (Bemis) to prevent desiccation, and it was inserted into a glass beaker filled with polystyrene pieces to keep the branch upright. While there is the potential for air-seeding to occur at the cut end using this methodology if we are not careful, recent work has shown that wrapping in Parafilm did not differ from keeping the stems submerged in water during the heat treatments (Salladay and Pittermann 2023). The branch was then placed in a convection oven, set at 100 °C, for 6 min in order to simulate exposure to a heat plume. The control branches were treated in the same manner but were not exposed to the simulated heat plume.

**Figure 1:**
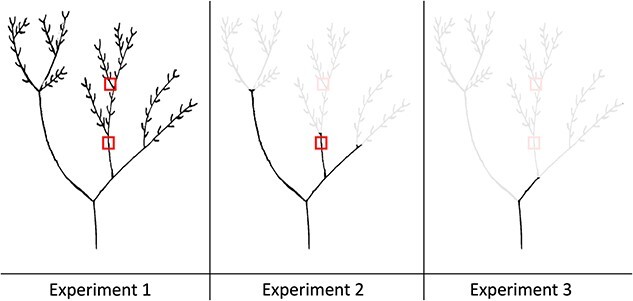
Schematic drawing of a generic branch for each experiment where the branch was sequentially trimmed back. In Experiment 1, the branch was only defoliated. In Experiment 2, the branch was trimmed back by removing the non-suberized sections of the branch. In Experiment 3, the branch was trimmed even further back to the next branch point. The red blocks indicate the non-suberized and suberized sections that were captured using the optical technique.

Following the heat-plume (or control) treatment, we ran three experiments (Figure 1) designed to determine if any embolism experienced in the branch was evenly distributed along its length or was localized near the distal tips. Each experiment was conducted on a new set of branches. Thus, each individual branch was only measured twice (post-treatment and flushed).

#### Experiment 1

Following the heat-plume treatment, both treated and control branches were defoliated under water by removing the leaf lamina at its junction with the petiole, and 2 cm of stem was cut from the base of the stem under water. Xylem conductance was then measured on the defoliated branches following the methods described in Kolb et al. (1996) and West et al. (2016). Briefly, branch samples were individually connected to an airtight sealed tube connected to a beaker containing 0.01 M KCl solution, filtered to 0.22 μm and placed on a balance (Metler Toledo). The branch was then placed in a cylindrical vacuum chamber, where a partial vacuum was created, pulling KCl solution from the reservoir beaker on the balance through the xylem (Kolb et al. 1996). Steady-state flow rate was then logged at a variety of pressures, and the slope of the flow rate and pressure was used to determine the hydraulic conductance (kg s^−1^ MPa^−1^). Following initial measurements, branches were flushed with 0.22 μm of filtered, 0.01 M KCl at 175 kPa for 1 h, and the hydraulic conductance was remeasured. Sapwood-specific stem hydraulic conductance, *K*_stem_ (mmol cm^−2^ s^−1^ MPa^−1^), was obtained by dividing the hydraulic conductance by the cross-sectional area of the xylem at the base of the shoot (cm^2^).

#### Experiment 2

Branches were treated as for Experiment 1, but in addition, prior to the measurement of hydraulic conductance, the youngest, non-suberized distal tips of the branch were excised (Figure 1). The impact of the heat plume on xylem conductance, excluding the distal tips, could thus be examined.

#### Experiment 3

Branches were treated as for Experiment 2 but were further trimmed back to the next branching point (Figure 1). The impact of the heat plume on xylem conductance, excluding the most distal node, could thus be examined.

#### Experiment 4

In Experiment 3, trimming the distal node resulted in the length of the branch being much less than the species maximum xylem vessel length. We conducted Experiment 4 to test whether our results for Experiment 3 may have been compromised by refilling of open vessels under vacuum infiltration. Branches were trimmed as for Experiment 3; however, instead of a simulated heat-plume treatment, the proximal end of the treatment branches was connected to a tube supplying N_2_ gas at 20 kPa, and the distal end was submerged in water. The presence of bubbles emerging from the submerged distal end confirmed the presence of open vessels in these segments and that these had been filled with air by our N_2_ treatment. Xylem conductance was then measured on these branches following the protocol described in previous experiments. We assumed that if refilling of open vessels occurred under our measurement protocol, then there would be little difference in conductance between the N_2_-treated branches and the same branches after being flushed with KCl. By contrast, a substantial difference between these measurements would indicate that open vessels did not refill under our measurement protocol and thus would not compromise the results of Experiment 3.

### Percentage loss of conductance

The PLC was calculated to compare treatment effects between the experiments using the following equation:


(1) \begin{equation*} \mathrm{PLC}=\frac{K_{\mathrm{stem}\_\mathrm{T}(\mathrm{F})}-{K}_{\mathrm{stem}\_\mathrm{T}}}{K_{\mathrm{stem}\_\mathrm{T}\left(\mathrm{F}\right)}}\times 100, \end{equation*}

where *K*_stem_T_ was the sapwood-specific conductance for the treatment branches and *K*_stem_T(F)_ was the sapwood-specific conductance for the flushed treatment branches.

### Relative changes in conductance

We compared sapwood-specific conductance of the control, unflushed branches (*K*_stem_C_) in Experiments 1–3 to determine if there was a difference in resistance segmentation between the species. We assumed that a greater relative increase in conductance from Experiments 1–3 would indicate a larger proportion of resistance in the distal parts of the branch and thus can be evidence of greater resistance segmentation. This relative change in conductance (*R*) was calculated for each experiment as:


(2) \begin{equation*} R= \frac{\overline{K_{\mathrm{stem\_C}}}}{\overline{K_{{\mathrm{stem\_C\_Exp1}}}}}\end{equation*}

where *K*_stem_C_Exp1_ is *K*_stem_C_ for Experiment 1. Error (*ε*) was propagated as:


(3) \begin{equation*} \varepsilon =R\times \sqrt{{\left(\frac{\varepsilon_{\overline{K_{\mathrm{stem}\_\mathrm{C}}}}}{\overline{K_{\mathrm{stem}\_\mathrm{C}}}}\right)}^2+{\left(\frac{\varepsilon_{\overline{K_{\mathrm{stem}\_\mathrm{C}\_\mathrm{Exp}1}}}}{\overline{K_{\mathrm{stem}\_\mathrm{C}\_\mathrm{Exp}1}}}\right)}^2} \end{equation*}

### Vulnerability to embolism of branch xylem

We determined the vulnerability to embolism of branch xylem for both species using the optical technique (Brodribb et al. 2016, 2017). Full details, including an overview of the technique and image processing, as well as scripts for step-by-step image capture and analysis, are available at http://www.opensourceov.org. Briefly, field-collected branches, which were not subjected to a heat-plume treatment, were re-cut underwater in the lab, with a minimum of at least 65 cm trimmed from the cut end, and the cut end of the branch was sealed with high vacuum grease (Dow Corning) and Parafilm (Bemis) to prevent excess moisture loss. Two small sections of the xylem were carefully exposed: a distal, non-suberized part of the branch and a suberized part ~10 cm further down the branch (Figure 1). Stem diameters for these sections were 2.3 ± 0.15 and 4.32 ± 0.19 mm, respectively. A flatbed scanner (Epson Perfection V800) was used to capture images of the exposed xylem of both sections of each plant using reflective mode, allowing for the observation of embolism within outer layers of xylem in each section of each stem. Briefly, the branch was placed on the scanner and was then taped to ensure it did not move while scanning.

Further down the branch, a larger section of bark was removed, and this section was >60 cm away from the cut end of the branch. A stem psychrometer (ICT PSY1) was fitted connected to the exposed region of xylem, sealed with high vacuum grease (Dow Corning). Stem xylem water potential was recorded every 10 min throughout the setup phase and for the duration of the scanning process. While the psychrometer was equilibrating, or during times of psychrometer failure, water potential was measured using Scholander-type pressure chamber (PMS instruments). The combinations of psychrometer and pressure chamber measurements were used to model the change in branch water potential with time (Figure S1 available as Supplementary data at *Tree Physiology* Online).

The branch was left to dry down for several days, ensuring all embolism events were captured during this time. Once image capture was completed, the image sequences were processed. The images were downloaded and were loaded onto Fiji ImageJ (National Institutes of Health) as an Image sequence. Each image was compared with each subsequent image to generate a pixel difference between successive images, revealing when the reflectance of light changed from xylem that was water-filled to xylem that was air-filled.

Vulnerability curves were fitted using a sigmoid function (Pammenter et al. 1998, Eq. (5)) using the nonlinear least square function in R (R Core Team 2018). This modeled the relationship between the percentage of embolized area and water potential for each section as:


(4) \begin{equation*} \mathrm{PLC}=\frac{100}{1+{e}^{\left(a\ast \left(x\ -\ b\right)\right)}}, \end{equation*}

where *a* relates to the steepness of the curve and *b* is the water potential (MPa) at 50% loss of conductance. The pressure at 12% loss of conductance (*P*_12_), 50% loss of conductance (*P*_50_) and 88% loss of conductance (*P*_88_) were also extracted from these following the procedures of Domec and Gartner (2001). The 95% confidence limits were obtained by bootstrapping.

### Statistical analysis

Differences between treatments were tested by ANOVA followed by post hoc Tukey tests. Differences between paired flushed and unflushed branches within each experiment were tested by paired *t*-tests. Differences in *P*_12_, *P*_50_ and *P*_88_ between non-suberized and suberized sections of each branch were tested by paired *t*-tests. All statistical analyses were conducted in R statistical software (R Core Team 2018).

## Results

### Xylem conductance

**Figure 2:**
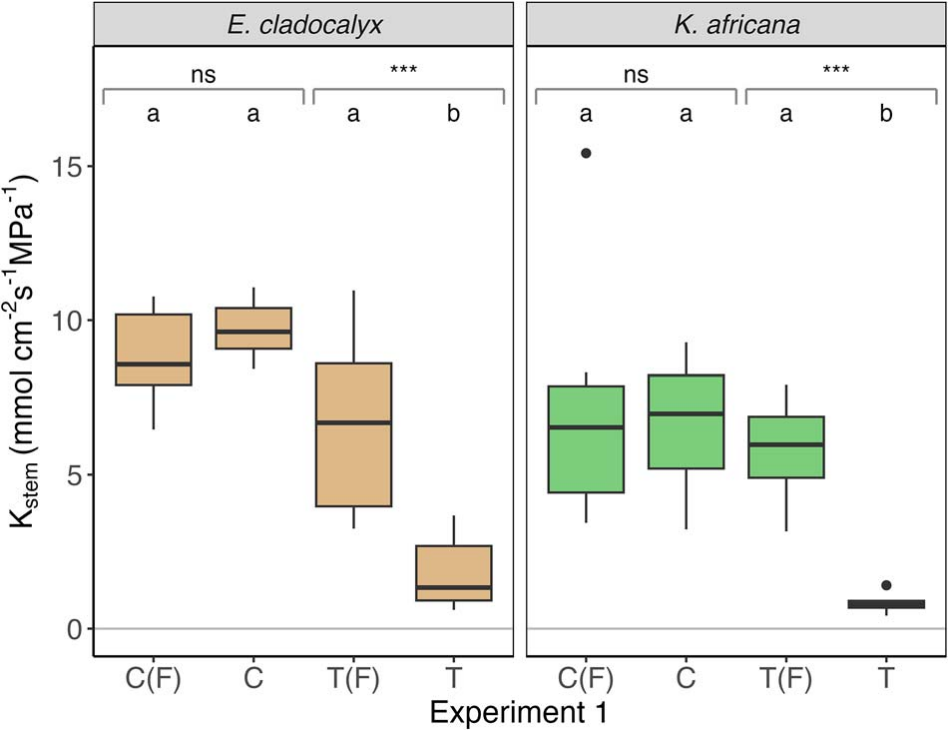
Experiment 1: sapwood-specific hydraulic conductance (*K*_stem_) of defoliated shoots of *E. cladocalyx* and *K. africana*. C(F) = ‘control flushed’, C = ‘control’, T(F) = ‘treatment flushed’ and T = ‘treatment’. Paired *t*-tests were conducted by comparing the treated and flushed conductance of the branches; the significant differences are indicated as ^*^^*^^*^P < 0.001 and ns is not significant. Treatments labeled with a common letter are not significantly different (ANOVA and post hoc Tukey HSD).

**Figure 3:**
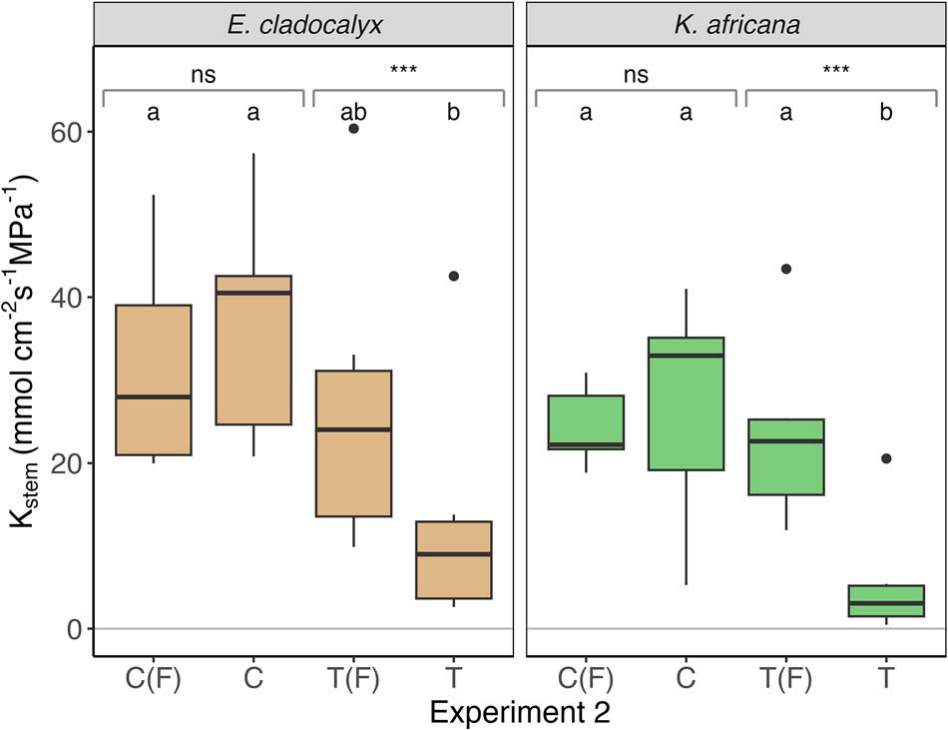
Experiment 2: sapwood-specific hydraulic conductance (*K*_stem_) of branches trimmed of non-suberized shoots for *E. cladocalyx* and *K. africana*. C(F) = ‘control flushed’, C = ‘control’, T(F) = ‘treatment flushed’ and T = ‘treatment’. Paired *t*-tests were conducted by comparing the treated and flushed conductance of the branches; the significant differences are indicated as ^*^^*^^*^P < 0.001 and ns is not significant. Treatments labeled with a common letter are not significantly different (ANOVA and post hoc Tukey HSD).

**Figure 4:**
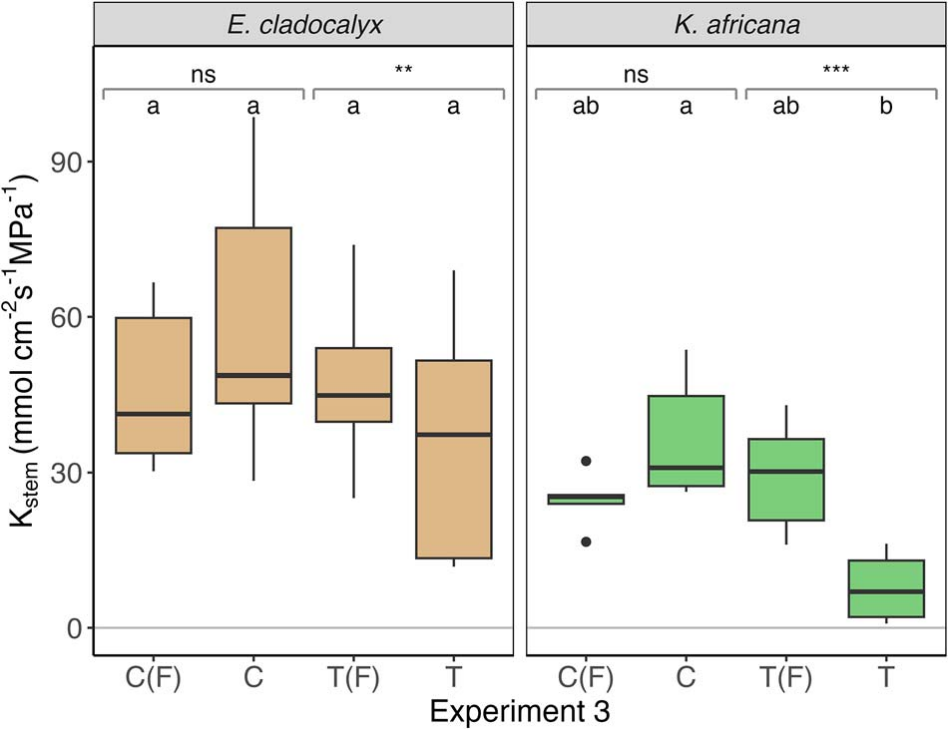
Experiment 3: sapwood-specific hydraulic conductance (*K*_stem_) of branches trimmed back a branch point for *E. cladocalyx* and *K. africana*. C(F) = ‘control flushed’, C = ‘control’, T(F) = ‘treatment flushed’ and T = ‘treatment’. Paired *t*-tests were conducted by comparing the treated and flushed conductance of the branches; the significant differences are indicated as ^*^^*^^*^P < 0.001, ^*^^*^P < 0.01 and ns is not significant. Treatments labeled with a common letter are not significantly different (ANOVA and post hoc Tukey HSD).

There was no difference in *K*_stem_ between control, control flushed and treatment flushed for either *E. cladocalyx* or *K. africana* (Table 1, Figures 2–4), indicating that (i) our field sampling protocol did not induce any extraneous embolism into the xylem, (ii) the plants were fully conductive prior to sampling and (iii) our flushing protocol was capable of returning the initial *K*_stem_ values post-treatment. The simulated heat plume resulted in a reduced *K*_stem_ for *E. cladocalyx* and *K. africana* for Experiment 1 (non-trimmed whole shoot) and Experiment 2 (non-suberized tips trimmed). However, only *K. africana* still experienced a reduced *K*_stem_ post-heat plume for Experiment 3 (full node trimmed), while *E. cladocalyx* did not (Table 2; Figures 2–4). There was no difference between the species in the relative change of *K*_stem_ in unflushed control branches with trimming (Figure S2 available as Supplementary data at *Tree Physiology* Online).

**Table 1:** Results for paired *t*-tests comparing the effect of flushing on control and treatment branches.

		Control branches			Heat-treated branches		
Species	Experiment	*t*	*df*	*P*	*t*	*df*	*P*
*E. cladocalyx*	1	1.84	5	0.938	−5.07	5	**0.002**
	2	1.52	6	0.910	−6.96	6	**<0.001**
	3	3.07	7	0.991	−2.92	7	**0.011**
*K. africana*	1	−0.64	5	0.276	−6.67	5	**0.001**
	2	0.58	5	0.707	−8.95	5	**<0.001**
	3	3.12	4	0.982	−5.74	5	**0.001**

Significant differences (*P* < 0.05) are highlighted in bold. Flushing had no effect on *K*_stem_ for control branches, but it was highly significant for heat-treated branches for all experiments in both species.

**Table 2:** Results from ANOVA testing for differences in *K*_stem_ between all treatments (control, heat-treated, flushed and unflushed) in each experiment for both species (see Figures 2–4).

Species	Experiment	*F*	*df*	*P*
*E. cladocalyx*	1	19.55	3,20	**<0.001**
	2	3.43	3,24	**0.033**
	3	1.66	3,28	0.198
*K. africana*	1	7.55	3,20	**0.001**
	2	5.73	3,20	**0.005**
	3	10.18	3,18	**<0.001**

Significant differences (*P* < 0.05) are highlighted in bold.

### Percentage loss of conductance

**Figure 5:**
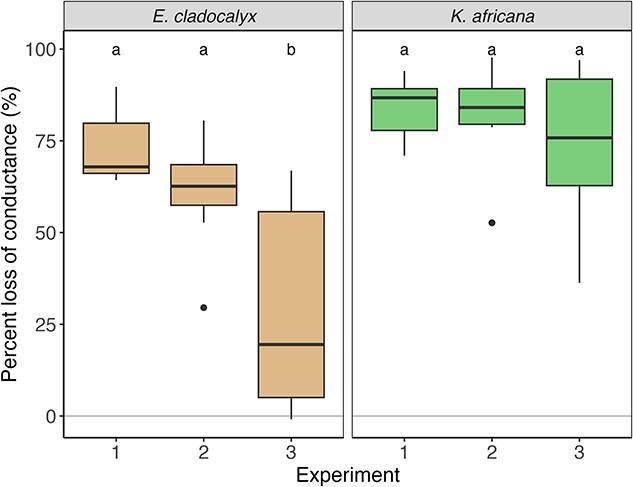
The PLC in *E. cladocalyx* and *K. africana* across all three heat-plumed experiments. Treatments labeled with a common letter are not significantly different (ANOVA and post hoc Tukey HSD tests).

The *E. cladocalyx* had 73% conductance loss in Experiment 1, 61% loss in Experiment 2 branches and 29% loss in the Experiment 3 branches (ANOVA: *E. cladocalyx*: *F* = 8.41, *df* = 2,18, *P* = 0.003; Figure 5). This implies that most embolism occurred in the distal part of the branch. Contrastingly, *K. africana* had 84% conductance loss in Experiment 1, 81% loss in Experiment 2 and 73% loss in Experiment 3 (ANOVA: *K. africana*: *F* = 0.618, *df* = 2,15, *P* = 0.55; Figure 5). This implies that embolism occurred throughout the branch and was not localized to the distal parts of the branch.

### Impact of open vessels

**Figure 6:**
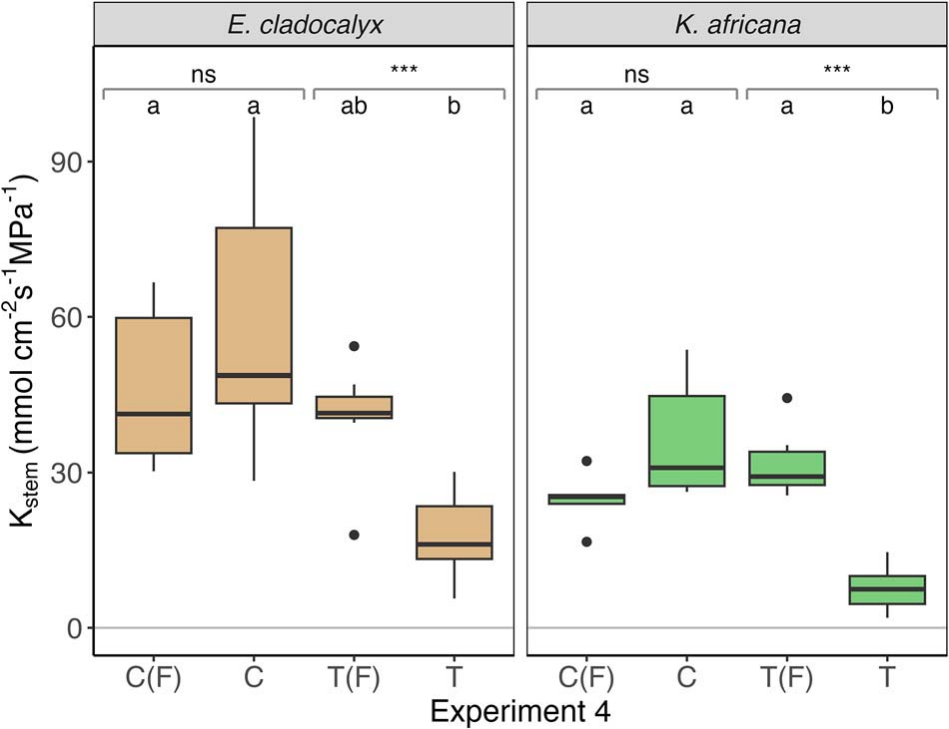
Open vessel experiment: sapwood-specific leaf conductance (*K*_stem_) for branches trimmed back by a full node of *E. cladocalyx* and *K. africana* and subjected to a low pressure (20 KPa) air treatment. C(F) = ‘control flushed’, C = ‘control’, T(F) = ‘treatment flushed’ and T = ‘treatment’. Paired *t*-tests were conducted comparing the treated and flushed conductance of the branches; the significant differences are indicated as ^*^^*^^*^P < 0.001 and ns is not significant. Treatments labeled with a common letter are not significantly different (ANOVA and post hoc Tukey HSD).

The possibility of refilling open vessels in Experiment 3, leading to an artificially low PLC in the treated branches, was a concern. However, this was not the case for both species, as blowing low pressure N_2_ gas into the treatment stems (i.e., air-filling open vessels) resulted in a measurable loss of conductance that was only regained upon flushing (Figure 6). For *K. africana*, heat-treated branches were statistically different to their flushed as well as the controls of the experiment (ANOVA: *K. africana*: *F =* 15.7, *df* = 3,18, *P* < 0.001; Figure 6). The *E. cladocalyx* heat-treated branches were not significantly different from the treatment flushed but were different from the controls (ANOVA: *E. cladocalyx*: *F* = 7.87, *df* = 3,28, *P* < 0.01; Figure 6). However, paired *t*-tests revealed a significant loss of conductance in treated branches that was recovered by flushing (paired *t*-test: *E. cladocalyx*: *t* = −8.75, *df* = 6, *P* < 0.001; *K. africana*: *t* = −18.46, *df* = 5, *P* < 0.001; Figure 6). This indicated that we were not refilling open vessels during measurements but were successfully measuring the loss of conductance in the branches.

### Vulnerability curves

**Figure 7:**
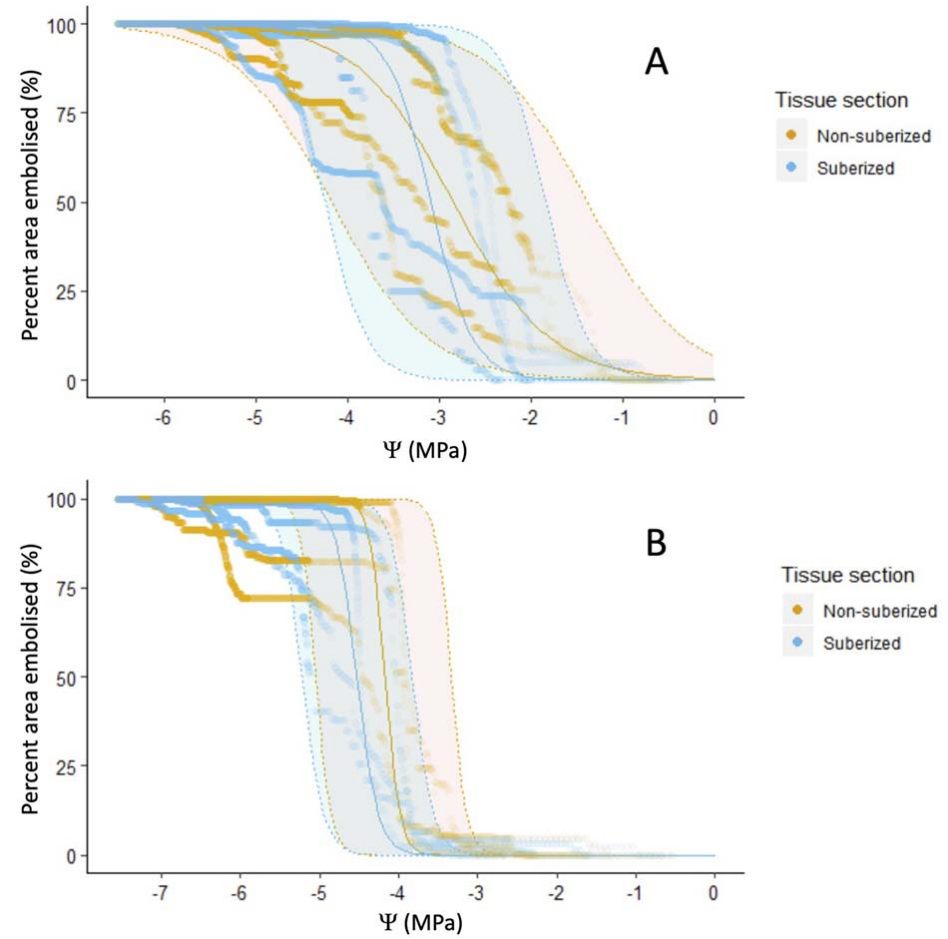
Relationship between the percentage of embolized area and the xylem water potential (Ψ) for the four samples of each species, (A) *E. cladocalyx* and (B) *K. africana* of the two different tissue sections, non-suberized and suberized, that were imaged. Modelled fits are sigmoid curves with 95% confidence intervals.

There were no strong differences in vulnerability to embolism between the suberized and non-suberized branch sections in *K. africana*. While the suberized section appeared to be slightly more resistant to embolism, this difference was not supported statistically with *P*_12_, *P*_50_ and *P*_88_, all statistically indistinguishable between the branch sections (Table 3; Figure 7). However, for *E. cladocalyx*, although *P*_50_ and *P*_88_ did not differ between the non-suberized and suberized sections, *P*_12_ was significantly higher in the non-suberized section than the suberized section (Table 3; Figure 7), indicative of a reduced air-entry point for embolism in the distal shoot for this species.

**Table 3:** The mean ± standard error of the pressures at 12% loss of conductance (P_12_), 50% loss of conductance (P_50_), and 88% loss of conductance (P_88_) for each tissue section (non-suberized and suberized) of each species. Results from paired t-tests comparing the non-suberized and suberized sections of the branch, are shown below the means.

	*E. cladocalyx*		*K. africana*	
	Non-suberized	Suberized	Non-suberized	Suberized
*P*_12_ (MPa)	−1.76 ± 0.26	−2.41 ± 0.25	−3.32 ± 0.27	−3.99 ± 0.12
	*t* = 10.54, *df* = 3, ***P***** < 0.001**		*t* = 2.25, *df* = 3, *P* > 0.05	
*P*_50_ (MPa)	−2.85 ± 0.33	−3.10 ± 0.31	−4.17 ± 0.22	−4.53 ± 0.18
	*t* = −0.62, *df* = 3, *P* > 0.1		*t* = 1.94, *df* = 3, *P* > 0.05	
*P*_88_ (MPa)	−3.94 ± 0.43	−3.79 ± 0.55	−5.02 ± 0.55	−5.07 ± 0.38
	*t* = −0.21, *df* = 3, *P* > 0.05		*t* = 0.21, *df* = 3, *P* > 0.1	

## Discussion

We tested whether the fire-tolerant *E. cladocalyx* displayed a greater degree of hydraulic segmentation when exposed to an embolism-inducing heat plume than the fire-intolerant *K. africana*. If this were the case, it would lend support to the hypothesis that hydraulic segmentation is an important trait in protecting the majority of a tree’s hydraulic continuum from hydraulic failure during fires. Our results, albeit limited to one fire-tolerant and one fire-sensitive species, were consistent with this hypothesis. The whole shoots of both *E. cladocalyx* and *K. africana* suffered a severe loss of conductance (c. 80%) when exposed to a simulated 100 °C heat plume (Figure 5), consistent with the findings of West et al. (2016). However, for *E. cladocalyx*, embolism was localized to the more distal ends of the shoot, with the PLC for the more basal branch segment less than half of that measured in the whole branch (Figure 5). By contrast, *K. africana* showed ±76% loss of conductance throughout the shoot (Figure 5), with no evidence of hydraulic segmentation. Thus, following our simulated heat plume, *E. cladocalyx* emerged with less than half the PLC in the basal parts of the shoot that *K. africana*.

Our second hypothesis was that hydraulic segmentation would be caused by differences in xylem vulnerability to embolism along the branch (i.e., ‘vulnerability segmentation’). Our results provided no support for a difference in resistance segmentation between the two species (Figure S1 available as Supplementary data at *Tree Physiology* Online), suggesting that vulnerability segmentation might be responsible for the observed patterns in PLC (Figure 5). A difference in vulnerability to embolism along the hydraulic continuum has been documented in conifer tree species, with the mature trunk being more vulnerable than the branches of the tree and with the shoots also having a high vulnerability (McCulloh et al. 2014). Evidence has been found for grapevines that had differing hydraulic traits along a single growing season (Sorek et al. 2021), thus adjusting vulnerability to embolism may have its trade-offs. Our results indicated that there were no easily detectable differences in vulnerability to embolism between the two distal-most sections of the branches for both species, with the exception of *P*_12_ for *E. cladocalyx*, where the non-suberized distal tips had a greater *P*_12_ than the more basal segments (Figure 7, Table 3). While this represents somewhat equivocal evidence for clear vulnerability segmentation, it is consistent with the hypothesis, as the presence of a higher *P*_12_ in the distal xylem of *E. cladocalyx* would result in earlier air entry into the xylem during a heat plume, inducing a drop in conductance and steepening the water potential gradient across this tissue, resulting in greater embolism. However, further work, including greater replication and assessment of vulnerability in the basal-most segments, is required before firm conclusions can be drawn on this mechanism.

We tested for two possible artifacts that could have influenced our results; namely excision and open vessel artifacts (Wheeler et al. 2013, Hacke et al. 2015). Embolism may result from cutting while xylem vessels are under tension (Wheeler et al. 2013). Our sampling protocol eliminated excision-embolism from the distal shoots evidenced by our control and control-flushed stem segments having similar conductance in all experiments. We also tested for the potential refilling of open vessels in trimmed branches, as this might lead to an under-estimation of embolism and a false detection of hydraulic segmentation. Our tests in Experiment 4 (Figure 6) indicated no refilling of open vessels, giving us confidence in our estimates of PLC from trimmed branches and our detection of hydraulic segmentation in *E. cladocalyx*.

Hydraulic segmentation should be beneficial for long-lived plants that experience potentially lethal droughts and fires, as it should result in the protection of a significant part of their hydraulic system from failure. Coupling hydraulic segmentation with the ability to resprout from distal buds should enable trees to recover rapidly post-fire. The *E. cladocalyx* has been shown to respond well to high-frequency fire (Ruthrof et al. 2003). For this species, the ability to resprout from live buds that are connected to a largely intact hydraulic system, protected by hydraulic segmentation, may explain its ability to rapidly recover its canopy post-fire. We speculate that high rates of canopy recovery post-fire may provide a good indication that a plant exhibits hydraulic segmentation as a pyrohydraulic trait.

By contrast, *K. africana* showed no hydraulic segmentation along the branch segment. The lack of hydraulic segmentation in this species is curious, as it suggests a cost to hydraulic segmentation. The *K. africana* is an Afromontane forest tree rarely subjected to drought or fire. We speculate that hydraulic segmentation may be maladaptive under conditions of little drought stress or fire, as it could potentially result in unnecessary loss of conductance in the distal tips, with possible leaf loss, under transient or mild drought conditions. The butterfly species, *Acraea horta*, is attracted to *K. africana* and their caterpillars often eat the tree bare of leaves (van Wyk 1997). Avoiding leaf loss through any transient or mild drought conditions early in the growing season would maximize carbon gain before herbivory, while the reduced leaf area post-herbivory would alleviate the need for hydraulic segmentation to protect the tree from late-summer drought. Lastly, *K. africana* does not resprout from fire-damaged portions of the crown. As such, there may be no selective pressure for hydraulic segmentation to protect the more basal parts of the hydraulic pathway through a fire, as there would be no new growth post-fire requiring a water supply.

The evidence for hydraulic segmentation in other plants is varied. Several studies have shown leaves to act as a hydraulic fuse (Tyree and Ewers 1991, Chen et al. 2009, 2010, Bucci et al. 2012, 2013, McCulloh et al. 2012), but contrastingly a lack of segmentation has been seen between leaf and stem (Skelton et al. 2017, Smith-Martin et al. 2020) and between petiole and stem (Li et al. 2020) in some plant species. A lack of segmentation under drought conditions has also been observed (Skelton et al. 2017, 2018, Li et al. 2020).

In relation to fire, Hoffmann et al. (2021) inferred that hydraulic segmentation did not prevent acute water loss in cut shoots of *M. grandiflora* during experimental leaf combustion. Using excised distal stem segments, with the cut end placed in water, and subjected to varying intensity of fire, they showed that fire intensity and water loss corresponded, providing further experimental evidence that fire leads to rapid water loss potentially leading to hydraulic failure (e.g., Kavanagh et al. 2010, West et al. 2016). Interestingly, they showed that complete combustion of leaves resulted in considerable water uptake from the shoot, which continued for a period post-combustion. They reasoned that the high rates of water loss measured could only have been achieved through steep water potential gradients that would have resulted in hydraulic failure in the xylem (not measured directly). Despite this, water transport into the stem continued for a brief period post-combustion, suggesting that any embolism that occurred in the xylem did not immediately prevent the stem from further water uptake. It would be extremely interesting to use Hoffmann et al.’s (2021) novel methodology on longer stem segments, where the potential for open vessels is reduced, coupled with direct measurements of loss of hydraulic conductance along the stem, to fully explore the impact of hydraulic segmentation on water loss across a variety of species.

Drought and fire frequency are increasing in many parts of the world due to climate change (Fensham et al. 2003, Ruffault et al. 2018, Williams et al. 2019). Drought and fire share a complex relationship, and drought can drive an increase in fire frequency and intensity (Pausas and Keeley 2021) as well as an increase in the susceptibility of trees to fire (van Mantgem et al. 2018). Conversely, trees may be less able to survive drought post-fire (Bär et al. 2018). Understanding the combined effect of drought stress and fire is a crucial challenge to be solved (Nolan et al. 2020) if we are to be able to predict the consequences of climate change on tree-dominated ecosystems. A more thorough understanding of the hydraulic traits that influence both drought and fire survival of trees will aid in this task.

## Supplementary Material

Supplementary_material_tpad108

## Data Availability

Data are available on request.
