# Supplementary material for: Hydraulic segmentation explains differences in loss of branch conductance caused by fire
Source: Tree Physiol. 2023 Sep 6;43(12):2121–30. doi: 10.1093/treephys/tpad108 (PMC10714316; doi:10.1093/treephys/tpad108)
Supplement: Supplementary_material_tpad108 [file supplementary_material_tpad108.docx]

# Supplementary material


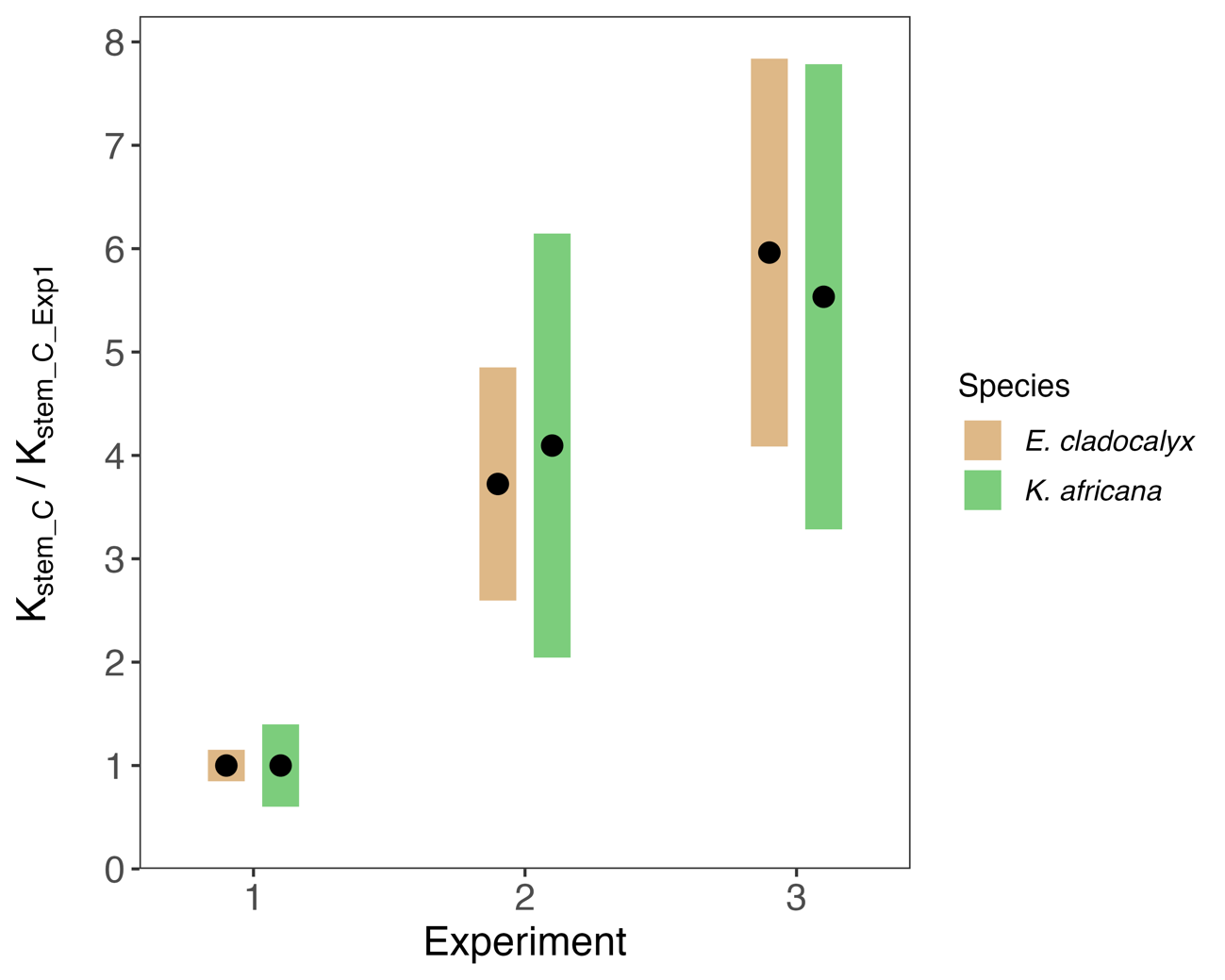


Figure S1: The ratio of sapwood-specific conductance of unflushed, control branches (K_stem_C_, mmol cm^-2^ s^-1^ MPa^-1^) relative to the values obtained in Experiment 1 (K_stem_C_Exp1_). This allows the interspecific comparison of how conductance increases as distal tissue is progressively removed (Experiment1 to Experiment 3). Both species show the same relative increase in conductance with trimming, indicating that there is little difference in how resistance is partitioned across these branches. This suggests no difference in resistance segmentation between these species.


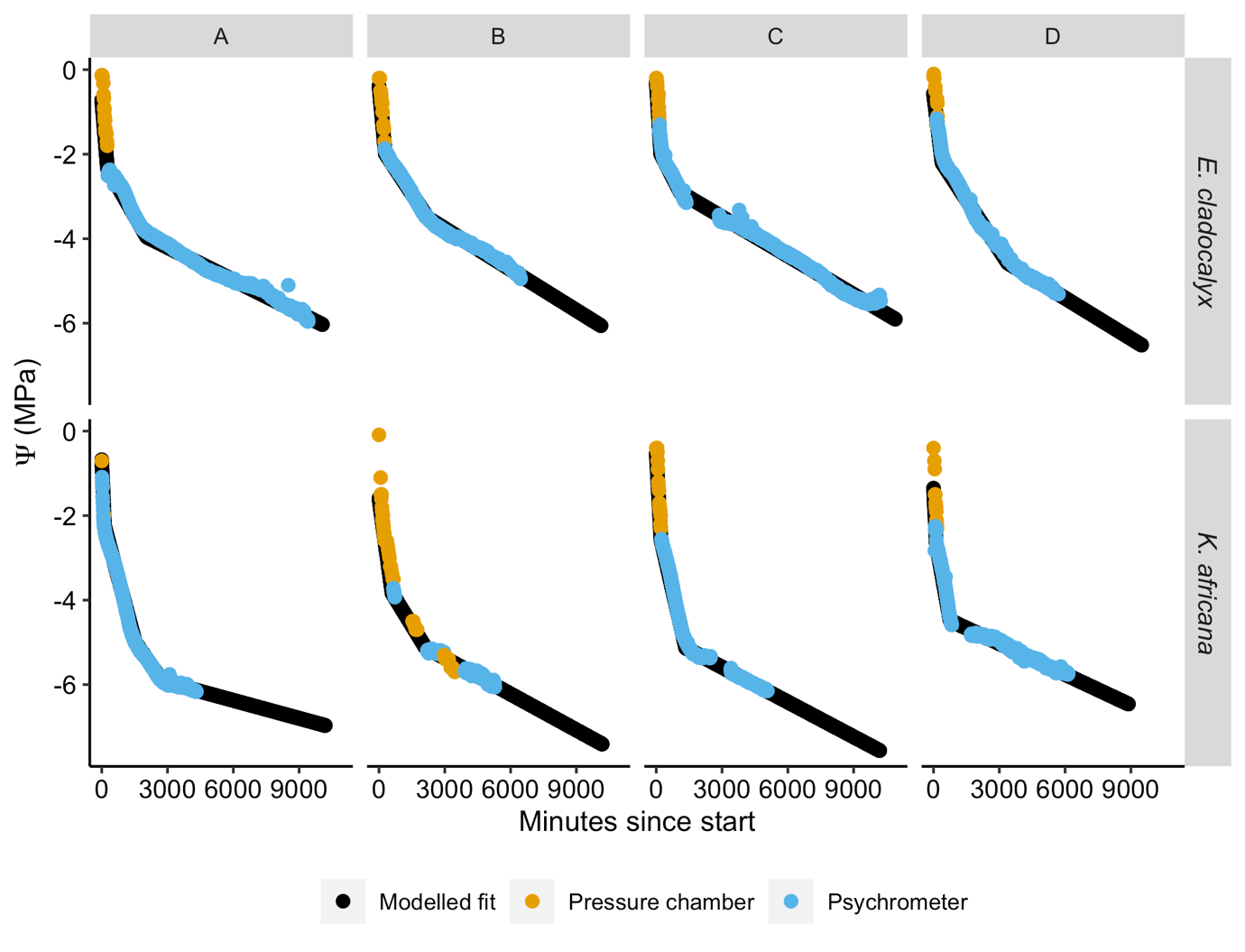


Figure S2: Branch water potential versus time since the start of the dry-down for the optical vulnerability curves. Water potential for each branch (A-D) of each species, *E. cladocalyx* (top) and *K. africana* (bottom), was measured using a pressure chamber (orange) and a stem psychrometer (blue). The relationship between water potential and time was modelled as three linear segments (black). The final segment was extrapolated to match the end of the optical data collection.
